# Enhanced p62-NRF2 Feedback Loop due to Impaired Autophagic Flux Contributes to Arsenic-Induced Malignant Transformation of Human Keratinocytes

**DOI:** 10.1155/2019/1038932

**Published:** 2019-10-30

**Authors:** Xiafang Wu, Ru Sun, Huihui Wang, Bei Yang, Fang Wang, Hongtao Xu, Shimin Chen, Rui Zhao, Jingbo Pi, Yuanyuan Xu

**Affiliations:** ^1^School of Public Health, China Medical University, China; ^2^The First Hospital of China Medical University, China; ^3^College of Basic Medical Sciences, China Medical University, China; ^4^School of Forensic Medicine, China Medical University, China

## Abstract

Chronic exposure to arsenic induces a variety of cancers, particularly in the skin. Autophagy is a highly conserved process which plays a dual role in tumorigenesis. In the present study, we found that chronic exposure to an environmentally relevant dose of arsenite induced malignant transformation of human keratinocytes (HaCaT) with dysregulated autophagy as indicated by an increased number of autophagosomes, activation of mTORC1 pathway, and elevated protein levels of p62 and LC3II. Meanwhile, arsenite-transformed cells showed lower intracellular levels of reactive oxygen species compared with control. Silencing *p62* ameliorated elevation in mRNA levels of NRF2 downstream genes (*AKR1C1* and *NQO1*) and malignant phenotypes (acquired invasiveness and anchor-independent growth) induced by chronic arsenite exposure. On the other hand, silencing *NRF2* abrogated the increase in mRNA and protein levels of p62 and malignant phenotypes induced by arsenite. In response to acute arsenite exposure, impaired autophagic flux with an increase in p62 protein level and interrupted autophagosome-lysosome fusion was observed. The increase in p62 protein levels in response to arsenite was not completely dependent on NRF2 activation and at least partially attributed to protein degradation. Our data indicate that accumulation of p62 by impaired autophagic flux is involved in the activation of NRF2 and contributes to skin tumorigenesis due to chronic arsenite exposure.

## 1. Introduction

Arsenic is a metalloid ubiquitously distributed in the environment. Chronic exposure to excessive levels of arsenic usually occurs through consumption of drinking water and contaminated food. Arsenic and arsenic compounds are identified as human carcinogens by the International Agency for Research on Cancer (IARC) [1]. Chronic exposure to arsenic induces a variety of cancers, particularly in the skin, lung, bladder, liver, and kidney [2]. However, the exact molecular mechanism of arsenic carcinogenicity is not well understood. The skin is one of the most sensitive tissues to chronic arsenic exposure. In humans, chronic exposure to arsenic results in various skin lesions, including hyperpigmentation, hyperkeratosis, and Bowen's disease, which are considered as precancerous lesions [3]. The characteristic arsenic-associated skin cancers include squamous cell carcinomas (SCCs) and basal cell carcinomas (BCCs) [4, 5].

Autophagy, an evolutionarily conserved cellular catabolic mechanism in eukaryotes, has vital roles in maintaining protein homeostasis and is essential to cell fate in response to stress [6]. Defects of autophagy lead to accumulation of dysfunctional organelles, damaged proteins, etc., which increase the risk of cancer [7, 8]. On the other hand, autophagy facilitates drug resistance and stress adaptation of cancer cells [9]. Thus, it is considered that autophagy suppresses tumor formation and growth in the early stage of cancer but promotes cancer in the later stage. p62 acts as an autophagy receptor and is usually degraded after autophagy with the use of lysosomal proteases [10, 11]. Elevated expression of p62 has been found in liver cancer, lung cancer, breast cancer, and skin cancer [1, 12–15]. Impaired autophagy resulting in p62 accumulation is reported to promote tumorigenesis [16]. Consistently, deficiency in *p62* diminishes chemical-induced hepatocarcinogenesis in the mouse model [14]. In skin tumors, p62 is upregulated and promotes cell proliferation and migration by stabilizing the oncogenic factor TWSIT1 [15].

It is interesting that p62 is able to form a positive feedback loop with nuclear factor erythroid 2-related factor 2 (NRF2) [17], a key transcription factor in antioxidative defense [18]. Accumulation of p62 inhibits Keap1-mediated NRF2 protein degradation by competing with NRF2 for the binding site of Keap1, resulting in transcriptional upregulation of NRF2 downstream genes [19, 20]. On the other hand, NRF2 regulates the expression of p62 by direct binding to the antioxidant response element on its promotor region. Our previous study has shown that NRF2 is constitutively activated in arsenic-transformed human keratinocytes (HaCaT cells) [21]. Recently, chronic exposure to low levels of arsenite has been found to inhibit autophagy [22–25], which is attributed to overproduction of interleukin 6 [23]. Moreover, NRF2 activation in the scenario of low-level arsenic exposure is indicated to be dependent on p62 accumulation due to blockage of autophagic flux rather than reactive oxygen species (ROS) [22, 25, 26]. However, the role of this p62-NRF2 feedback loop in arsenic carcinogenesis has not been clearly identified.

In the present study, we found that arsenite-transformed human keratinocytes showed dysregulated autophagy with enhanced p62-NRF2 feedback loop and decreased intracellular ROS levels. Acute exposure to the environmentally relevant dose of arsenite blocked autophagic flux by interfering autophagosome-lysosome fusion, which contributed to the accumulation of p62. Silencing *p62* or *NRF2* ameliorated the arsenite-induced enhancement of p62-NRF2 feedback loop and furthermore the acquisition of malignant phenotypes. Our data suggest the important role of p62-NRF2 feedback loop in arsenite-induced skin tumorigenesis. This loop may be a target in prevention and therapy of arsenite-induced skin cancer.

## 2. Results

### 2.1. Chronic Exposure to an Environmentally Relevant Dose of Arsenite Induces Malignant Transformation and Dysregulated Autophagy in Human Keratinocytes

**Figure 1:**
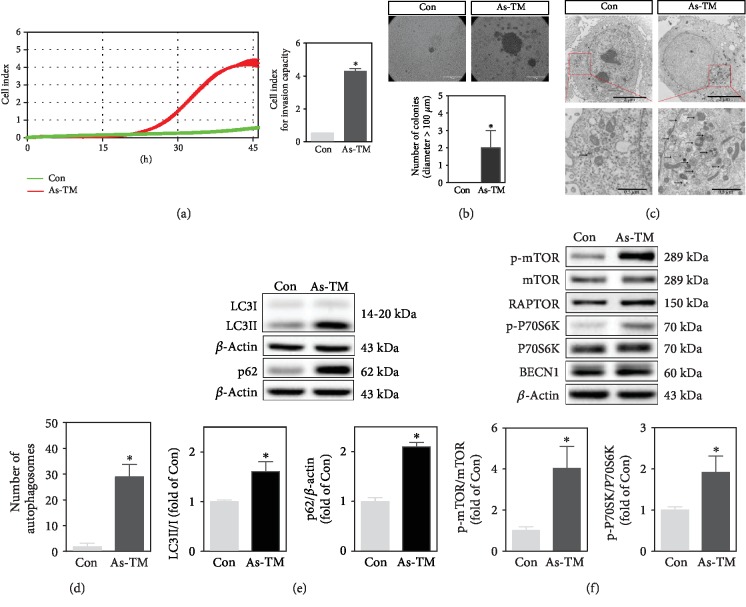
Alterations of autophagy markers and upstream signaling pathways in arsenite-transformed (As-TM) cells. HaCaT cells were treated with 100 nM of sodium arsenite (As) for 30 weeks. Passage-matched nontreated cells were used as the control (Con). (a) Cell invasion analyzed with real-time cell analysis (RTCA) xCELLigence system. The left chart shows the kinetic analysis of cell invasion. The right panel shows the average index reflecting cell invasion capacity at 45 h. (b) Colony formation in soft agar. Representative images of the control (upper left panel), As-TM cells (upper right panel), and quantification of colonies (lower panel) are shown. Scale bar is 100 *μ*m. For quantification, three fields of view were randomly selected from each 35 mm culture dish. The number of clones with a diameter greater than 100 *μ*m was counted. (c) Increase in number of autophagosomes in As-TM cells. Autophagosomes were observed with transmission electron microscopy (TEM). Scale bar is 2 *μ*m (up) and 0.5 *μ*m (down). Arrows indicate autophagosomes. (d) Number of autophagosomes per cell according to TEM. (e) Western blot for autophagy markers, LC3 and p62. Upper: representative image; lower: quantification of protein levels of LC3II/I and p62 determined with Western blot. (f) Western blot for BECN1 and proteins in the mTORC1 pathway, including p-mTOR, mTOR, RAPTOR, p-P70S6K, and P70S6K. Upper: representative image; lower: quantification of p-mTOR/mTOR and p-P70S6K/P70S6K determined with Western blot. *n* = 3 except for colony formation, in which *n* = 6. ^∗^*p* < 0.05, compared with Con.

After a 30-week continuous arsenite exposure, HaCaT cells exhibited increased invasion capacity (Figure 1(a)) and anchorage-independent growth as illustrated by the formation of bigger colonies in soft agar (Figure 1(b)) compared with control (Con), all indicative of malignant transformation [27]. Thus, the 30-week arsenite-exposed cells were named as arsenite-transformed (As-TM) cells thereafter. Transmission electron microscopy (TEM) analysis of As-TM cells showed the massive accumulation of autophagosomes (Figures 1(c) and 1(d)), recognized as double-membrane vesicles engulfing cytosolic contents or organelles. The ratio of LC3II to LC3I (LC3II/I) and protein levels of p62 were markedly increased in As-TM cells compared with the control (Figure 1(e)). Upstream signaling pathways of autophagy, such as mTORC1 and BECN1, were also determined. The levels of phosphorylated mTOR (p-mTOR) and phosphorylated P70S6K (p-P70S6K) were higher in As-TM cells than control (Figure 1(f)). No alteration was found in protein levels of BECN1 or RAPTOR in As-TM cells (Figure 1(f)). Collectively, these data indicate that chronic exposure to an environmentally relevant dose of arsenite induces malignant transformation of HaCaT cells with dysregulated autophagy.

### 2.2. Amplified p62-NRF2 Autoregulatory Loop Is Required for Arsenite-Induced Malignant Transformation of Human Keratinocytes

**Figure 2:**
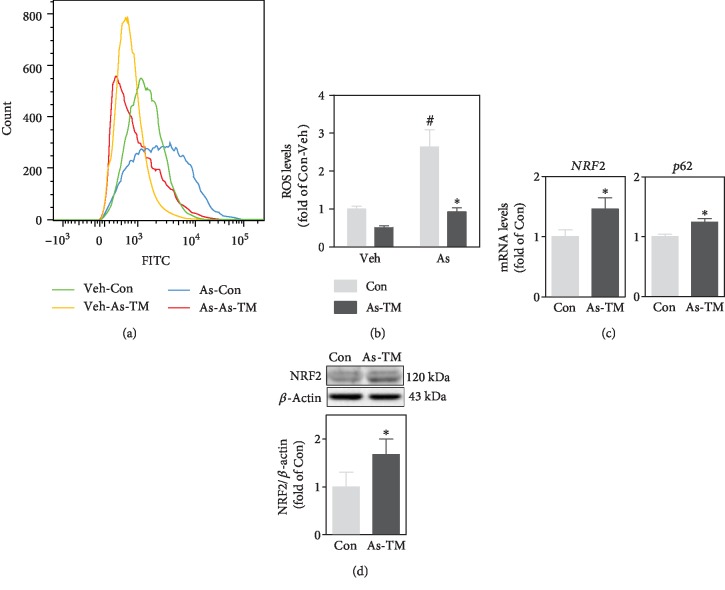
Long-term exposure to low-level arsenite induced adaptive antioxidative response in HaCaT cells. (a) Representative histogram for intracellular ROS levels detected by flow cytometer. As-TM cells or passage-matched nontreated control (Con) was challenged with 10 *μ*M of sodium arsenite (As) or equal volume of PBS (Veh) for 24 h. (b) Quantification of intracellular ROS levels determined by flow cytometer. (c) mRNA levels of NRF2 and p62 in As-TM and control cells. (d) Western blot of NRF2 in As-TM and control cells. Upper: representative image; lower: quantification of NRF2 protein levels determined with Western blot. *n* = 3. ^∗^*p* < 0.05, compared with Con compartment. ^#^*p* < 0.05, compared with Veh compartment.

Arsenite is a well-known oxidative stressor [28]. However, in As-TM cells, intracellular ROS levels were only 50% of the control (Figures 2(a) and 2(b)). When cells were acutely challenged by a relatively high dose (10 *μ*M) of arsenite, intracellular ROS levels were increased to 2.8-fold of Veh in control cells and 1.8-fold of Veh in As-TM cells, respectively (Figures 2(a) and 2(b)). The increase in ROS levels in As-TM cells was not as much as the control (*p* < 0.05) (Figures 2(a) and 2(b)). NRF2 and p62 were increased in both mRNA levels (Figure 2(c)) and protein levels (Figures 1(e) and 2(d)) in As-TM cells compared with the control (*p* < 0.05). This is consistent with our previous finding that NRF2 is activated in As-TM cells [21]. These results also indicate that adaptive antioxidative response instead of oxidative stress occurs in chronic arsenite-exposed cells.

**Figure 3:**
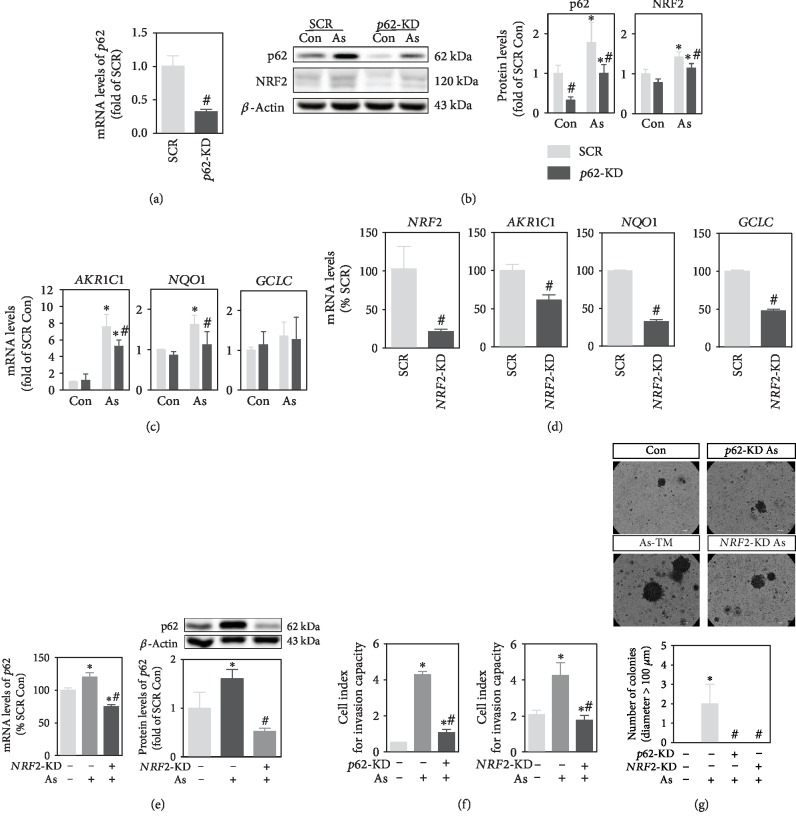
Amplification of p62-NRF2 feedback loop is required for the acquisition of arsenite-induced malignant phenotypes. (a) mRNA levels of *p62* in HaCaT cells infected with lentiviral vector expressing shRNA targeting *p62* (*p62*-KD) or scrambled nontarget negative control (SCR). (b) Protein levels of p62 and NRF2 in *p62*-KD and SCR cells. Left: representative image; right: quantification of protein levels of p62 and NRF2 determined with Western blot. (c) mRNA levels of *NRF2* downstream genes, *AKR1C1*, *GCLC*, and *NQO1*, in *p62*-KD and SCR cells. (d) mRNA levels of *NRF2* and its downstream genes in chronic arsenite-exposed cells with *NRF2* knockdown (*NRF2*-KD). (e) mRNA and protein levels of p62 in *NRF2*-KD cells analyzed with RT-PCR (left) and Western blot (right), respectively. Upper right: representative image for Western blot; lower right: quantification of p62 protein levels determined with Western blot. (f) Invasion capacity determined by xCELLigence RTCA. Cell index at 45 h after seeding was used to assess invasion capacity. (g) Colony formation in soft agar. Representative image (upper) and quantification of the colonies (lower). Scale bar is 100 *μ*m. As (As+): cells were chronically exposed to 100 nM of sodium arsenite for 30 weeks. Con: passage-matched nontreated cells. *n* = 3 except for colony formation assay, in which *n* = 6. ^∗^*p* < 0.05, compared with control (As-) compartment. ^#^*p* < 0.05, compared with SCR (*NRF2*-KD- or *p62*-KD-) compartment.

Amplified p62-NRF2 autoregulatory loop has been observed in several arsenic-exposed cells [22, 25]. However, the role of this loop in arsenic carcinogenesis is not fully defined. Thus, we silenced *p62* and *NRF2*, respectively, to determine whether suppression of this loop affects arsenic-induced malignant transformation. Successful knockdown of *p62* was verified at mRNA and protein levels (Figures 3(a) and 3(b)). The protein expression of NRF2 in total cell lysis, as well as downstream genes of *NRF2*, *AKR1C1*, and *NQO*1, was significantly suppressed by *p62* silencing in arsenite-exposed cells (Figures 3(b) and 3(c)). Effective *NRF2* silencing was verified by mRNA levels of itself and its downstream genes (Figure 3(d)). Silencing *NRF2* abolished arsenite-induced upregulation of p62 in both mRNA and protein levels (Figure 3(e)). Moreover, when cells with silenced *NRF2* or *p62* were exposed to arsenite for 30 weeks, the invasion capacity (Figure 3(f)) and colony-forming ability (Figure 3(g)) were significantly decreased compared with scramble.

### 2.3. Arsenite Exposure Blocks Autophagic Flux by Interfering Autophagosome-Lysosome Fusion

**Figure 4:**
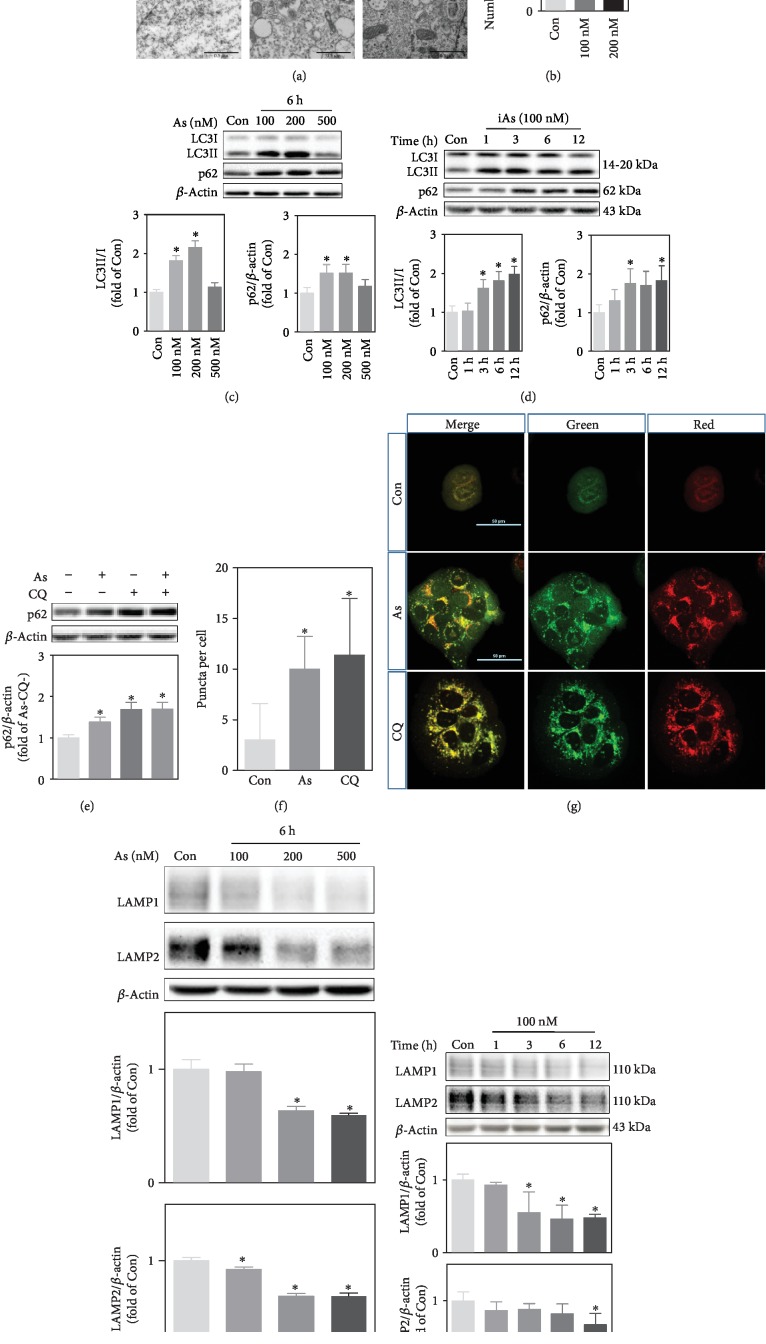
Arsenite inhibits autophagosome-lysosome fusion. (a) Autophagosomes observed with TEM in cells nontreated (Con) or treated with 100 nM or 200 nM sodium arsenite for 4 h. Arrows indicate autophagosomes. Scale bar is 2 *μ*m (up) and 0.5 *μ*m (down). (b) Number of autophagosomes per cell according to TEM. (c) Western blot for LC3 and p62 in HaCaT cells treated with 100 nM, 200 nM, or 500 nM arsenite for 6 h. (d) Western blot for LC3 and p62 in HaCaT cells treated with 100 nM arsenite at different time points. (e) Protein levels of p62 detected with Western blot. Arsenite-induced inhibition of autophagic flux was tested with chloroquine (CQ, 30 *μ*M) pretreatment and in the absence (-) or presence (+) of 100 nM arsenite for 6 h. (f) Quantification of orange/yellow LC3 puncta in the cell. HaCaT cells were transfected with a tandem mRFP-GFP-LC3 and then treated with 100 nM arsenite for 4 h or 30 *μ*M CQ for 6 h. The number of puncta in cells was counted using ImageJ software. Average number of orange/yellow puncta per cell from 16 randomly selected cells in each group was shown. (g) Representative image of LC3 fluorescence observed by a confocal microscope. Scale bar is 50 *μ*m. (h) Western blot for LAMP1 and LAMP2 in HaCaT cells treated with 100 nM, 200 nM, or 500 nM arsenite for 6 h. (i) Western blot for LAMP1 and LAMP2 in HaCaT cells exposed to 100 nM arsenite at different time points. For Western blot, upper: representative image; lower: quantification of protein levels determined with Western blot. *n* = 3. ^∗^*p* < 0.05, compared with Con (or As- and CQ-).

Amplified p62-NRF2 autoregulatory loop can be attributed to impaired autophagy flux [22]. To further investigate the specific alteration of autophagy in response to arsenite, HaCaT cells were treated with arsenite at the concentrations of 100 nM or 200 nM for 6 h. Consistent with the results in As-TM cells, accumulation of autolysosomes in arsenite-treated cells was observed (Figures 4(a) and 4(b)). Though arsenite led to a concentration-dependent increase in LC3II/I and p62 protein levels within 200 nM at 6 h, 500 nM arsenite did not show such an effect (Figure 4(c)). In the time-course analysis, protein levels of LC3II/I and p62 were significantly increased in cells treated with 100 nM of arsenite after 3 h (Figure 4(d)). Accumulation of p62 is an indicator for defects in autophagic degradation [29, 30]. To assess how arsenite interferes with autophagic flux, lysosomal protease inhibitor chloroquine (CQ) was used. The protein levels of p62 were not significantly changed in cells treated with CQ plus arsenite compared to cells treated with CQ alone (Figure 4(e)). These results indicate that the accumulation of p62 induced by arsenite is due to the blockage of autophagosome degradation.

We next compared the location of a tandem mouse red/green fluorescent protein- (mRFP-) GFP-LC3 signals to the lysosome, to determine whether arsenite impaired autophagosome-lysosome fusion. HaCaT cells transfected with mRFP-GFP-LC3 showed yellow/orange puncta due to CQ treatment (Figures 4(f) and 4(g)), which is known to inhibit autophagosome-lysosome fusion [31]. We found similar results in cells exposed to arsenite for 4 h with varying degrees of yellow/orange convergence (Figures 4(f) and 4(g)), indicating that arsenite impairs autophagic flux by preventing the fusion of autophagosomes and lysosomes. Lysosome-associated membrane protein (LAMP) 1 and LAMP2 are major protein components of the lysosomal membrane, which mediate a number of essential functions of this compartment [32]. A concentration (100 nM to 500 nM at 6 h)- and time (100nM within 12 h)- dependent reduction in protein levels of LAMP1 and LAMP2 in response to arsenite was observed (Figures 4(g) and 4(h), respectively).

### 2.4. P62 Overexpression Induced by Environmentally Relevant Dose of Arsenite Is Attributed to Enhanced Transcription and Reduced Protein Degradation

**Figure 5:**
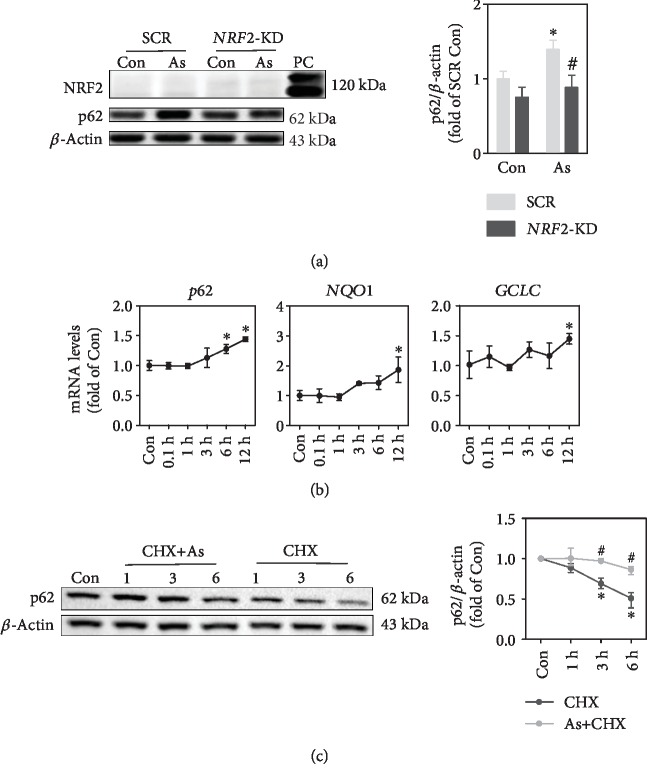
Enhanced transcription and decreased protein turnover contribute to accumulation of p62 protein in response to arsenite exposure. (a) Western blot of NRF2 and p62 under basal and arsenite-treated conditions in SCR and *NRF2*-KD cells. As: cells were treated with 100 nM sodium arsenite for 6 h. PC: positive control, cells were treated with 20 *μ*M sodium arsenite for 6 h. (b) mRNA levels of *p62*, *NQO1*, and *GCLC* in HaCaT cells treated with 100 nM sodium arsenite at different time points. (c) Exposure to arsenite inhibited p62 degradation in HaCaT cells. Cells were treated with CHX (10 *μ*g/mL) or CHX+ As (100 nM) at different time points, followed by Western blot analysis. For Western blot, left: representative image; right: quantification of p62 protein levels. *n* = 3. ^∗^*p* < 0.05, compared with Con compartment. ^#^*p* < 0.05, compared with SCR compartment or CHX-treated compartment.

Arsenite has been shown to activate NRF2, which transcriptionally regulates p62 expression. No alteration was found in NRF2 protein expression in total cell lysis at 6 h with 100 nM of arsenite exposure, while p62 protein levels were increased (Figure 5(a)). Analysis of *NRF2* downstream genes showed that mRNA levels of *p62* were significantly increased at 6 h, but mRNA levels of NQO1 and GCLC were upregulated at 12 h (Figure 5(b)). *NRF2* silencing suppressed arsenite-induced p62 overexpression (Figure 5(a)). As expected, arsenite, even at such a low nontoxic level, induces NRF2 activation which contributes to increased p62 protein levels. Further, we assessed the effect of arsenite on p62 turnover. As shown in Figure 5(c), inhibition of protein synthesis by cycloheximide (CHX) decreased p62 protein levels. Under such a condition, arsenite treatment significantly attenuated decrease of p62 protein levels (Figure 5(c)), indicating that the inhibition of protein degradation contributes to p62 accumulation by arsenite.

## 3. Materials and Methods

### 3.1. Cell Culture and Arsenite Exposure

HaCaT human keratinocytes (N.E. Fusening, German Cancer Research Center, Heidelberg, Germany) were cultured in Dulbecco's modified Eagle's medium (DMEM) (Thermo Fisher Scientific, Beijing, China) supplemented with 10% fetal bovine serum (FBS) (Biological Industries (BI), Hazafon, Israel) and 1% penicillin-streptomycin solution (BI) at 37°C in a humidified 5% CO_2_ atmosphere. Cells at 80% confluence were passaged according to 1 : 5 proportion. The culture medium was refreshed every 2 days. For chronic arsenite exposure, HaCaT cells were maintained continuously in a medium containing 100 nM of sodium arsenite (NaAsO_2_) (Sigma-Aldrich, St. Louis, USA) for 30 weeks. Passage-matched nontreated cells were used as the control. This arsenite concentration is comparable to the blood arsenite level of chronic arsenicosis patients [21, 33].

### 3.2. Lentiviral-Based shRNA Transduction

Transduction of HaCaT cells with lentiviral-based shRNAs targeting *NRF2* (SHVRS-NM 010902, Sigma-Aldrich), *p62* (GeneChem, Shanghai, China), or scrambled nontarget negative control was performed as described previously [34, 35]. The selection media for HaCaT cells contained 1 *μ*g/mL of puromycin (Thermo Fisher Scientific).

### 3.3. Kinetics of Cell Invasion

A cell invasion test was performed on the RTCA xCELLigence system (ACEA Biosciences Inc., CA, USA) equipped with a CIM-plate 16. The plate is composed of upper and lower chambers separated by a microporous metallic membrane with a thin layer of Matrigel basement membrane. In each well, 4 × 10^4^ cells were added in the upper chamber. The xCELLigence system based on electrical impedance measurement allows for the dynamic monitoring [36]. Electronic signals at the lower chamber as invasion cell indexes were monitored every min for up to 50 h. The cell index was calculated as follows: (impedance at time point *n*‐impedance in the absence of cells)/nominal impedance value [36].

### 3.4. Colony Formation in Soft Agar

We used a colony formation assay to assess anchorage-independent growth. 2 mL of 0.5% agar (Sigma-Aldrich) in complete growth media was used to cover the bottom of each 35 mm dish. 1.25 × 10^4^ cells suspended with 1 mL of 0.33% agar in complete growth media were overlaid onto base agar. Cells in agar medium were cultured in a CO_2_ incubator. The colonies were identified with iodonitrotetrazolium chloride (INT) (Sigma-Aldrich) staining after a 4-week incubation.

### 3.5. Transmission Electron Microscopy

Cell pellets were fixed in 2.5% glutaraldehyde solution (Sigma-Aldrich) in 0.1 M phosphate buffer for 24 h at 4°C, dehydrated using a gradient series of ethanol, and infiltrated with Epon 812 (Structure Probe, Inc., West Chester, USA). Finally, the specimens were cut into 1 *μ*m sections with LKB-V ultramicrotome (Bromma, Stockholm, Sweden) and stained with uranyl acetate and lead citrate. Sections were examined using a transmission electron microscope (Hitachi, Tokyo, Japan).

### 3.6. Reverse Transcriptase Quantitative Polymerase Chain Reaction (RT-qPCR)

RT-qPCR was conducted as previously described [37]. Total RNA was isolated using a TRIzol reagent (Thermo Fisher Scientific) and reverse transcribed to cDNA with a Prime Script RT reagent Kit (TaKaRa, Dalian, China). A SYBR Premix Ex Taq Kit (TaKaRa) and QuantStudio 6 Flex Real-Time PCR System (Applied Biosystems, Waltham, USA) were used to assess cDNA amplifications. Data were analyzed using the delta-delta cycle time (CT) method. All primers were designed with Primer-BLAST online (https://www.ncbi.nlm.nih.gov/tools/primer-blast) and obtained from Sigma-Aldrich. Primer sequences are provided in supplemental material [Supplementary-material supplementary-material-1]. The average CT value of *β*-actin and GAPDH was used as the value for internal control. The assays were conducted in triplicate for each sample, and three experiments from separately generated samples were performed.

### 3.7. Western Blot

Cells were incubated with cell lysis buffer (Cell Signaling, Danvers, USA) containing inhibitor cocktail of protease and phosphatase (Sigma-Aldrich) on ice for 30 min. The protein sample (25-40 *μ*g) was run on 6%, 10%, or 15% Tris-glycine gels, transferred to polyvinylidene fluoride (PVDF) membrane, blocked in 5% nonfat dry milk at room temperature (RT) for 2 h, and incubated with the primary antibody at 4°C overnight and secondary antibody at RT for 1 h. Membranes were developed with electrochemiluminescence (Tanon, Shanghai, China) and subsequently autoradiographied (Tanon). Quantification of the results adjusted to internal reference protein (*β*-actin) was performed by ImageJ (Standard Edition, Bethesda, USA). Primary antibodies for phospho-mTOR (#s6448, 1 : 1000), mTOR (#2983P, 1 : 1000), phospho-P70S6K (#9234, 1 : 1000), RAPTOR (#2280, 1 : 1500), P70S6K (#2708, 1 : 1000), and LAMP2 (#49067, 1 : 1000) were from Cell Signaling Technology. Primary antibodies for LC3 (#12135-1-AP, 1 : 1000), p62/SQSTM1 (#18420-1-AP, 1 : 10000), LAMP1 (#21997-1-AP, 1 : 1000), and BECN1 (#11306-1-AP, 1 : 1000) were from Proteintech Group (Wuhan, China). Primary antibodies for NRF2 (#SC13032, 1 : 500) and *β*-actin (#SC1616, 1 : 5000) were from Santa Cruz Biotechnology (Santa Cruz, USA). Secondary antibodies were from Thermo Fisher Scientific.

### 3.8. Intracellular ROS Measurement

ROS levels were measured by flow cytometry using 5-(and-6)-chloromethyl-2′,7′-dichlorodihydrofluorescein diacetate, acetyl ester (CM-H2DCFDA) (Thermo Fisher Scientific). Briefly, cells in each group were washed with PBS and incubated with 5 *μ*M of CM-H2DCFDA at RM in dark for 30 min. The fluorescence of dichlorofluorescein was measured using Canto II flow cytometry (Becton Dickinson, San Jose, USA) with an excitation wavelength of 488 nm and emission wavelength of 525 nm.

### 3.9. Autophagosome-Lysosome Fusion Detection

HaCaT cells were incubated with HBLV-mRFP-GFP-LC3 adenovirus (Hanhbio, Shanghai, China) for 24 h and screened with puromycin. The transduced cells were cultured in glass bottom cell culture dishes (NEST, Wuxi, China) and treated with 100 nM of sodium arsenite for 4 h or 30 *μ*M of CQ for 6 h. Pictures were acquired with AI^+^ confocal microscopy (Nikon, Tokyo, Japan).

### 3.10. Statistical Analysis

All statistical analyses were performed by using GraphPad Prism 5 software (La Jolla, USA). For comparison between two groups, a *t*-test was performed. For comparison among multiple groups, one-way ANOVA with Tukey's multiple comparison test or two-way ANOVA with the Bonferroni post hoc test was performed. *p* < 0.05 was considered significant. Data were expressed as mean ± standard deviation (SD). Each experiment was independently repeated for at least three times.

## 4. Discussion

Environmental arsenic exposure has been known as a risk factor for skin cancer according to a number of epidemiological studies [13, 38, 39]. So, it is important to elucidate the intracellular target in response to arsenic at low, environmentally relevant doses. Arsenic is well known to increase intracellular ROS levels when the exposure dose is high [40]. However, at a relatively low dose, arsenic may not significantly increase ROS levels. When human fibroblast cells were exposed to 500 nM of arsenite for 24 h, ROS levels significantly decreased compared with control due to induction of stress-responsive genes [41]. In the present study, treatment with 100 nM of arsenite did not significantly change ROS levels within 24 h (data not shown), but decreased ROS levels in the long term with enhanced p62-NRF2 loop. Similarly, acute exposure to arsenite (2 h or 4 h) below 10 *μ*M did not alter intracellular ROS amount in various cell lines, as detected with the same method in the present study or even with a more sensitive ROS detection method (electron paramagnetic resonance spectroscopy) [20, 26]. In this scenario, arsenic does not appear to activate NRF2 via excessive generation of ROS, though the subtle ROS changes beyond sensitivity of the detection method cannot be excluded. It has been demonstrated that arsenic does not activate NRF2 in the traditional Keap1-C151-depedent manner [42], which is common for transient NRF2 activation by sepharophone or tBHQ [20]. Earlier studies have established a noncanonical pathway for NRF2 activation mediated by p62 in arsenic-exposed cells [17, 20–22, 43]. Our data provide further evidence that ROS-independent noncanonical NRF2 activation may be involved in carcinogenesis caused by the environmentally relevant dose of arsenite.

Clearly, NRF2 protects cells from harmful effects of electrophilic and oxidative stressors [18]. It is considered that some NRF2 inducers, such as natural small molecules and food antioxidants, can reduce the risk of cancer. This concept is further verified in rodent experiments. *Nrf2*-deficient mice are more susceptible to chemical carcinogens [44, 45]. Although a transient increase in NRF2 levels is able to achieve protective antioxidant effects, constitutive NRF2 activation is very common in many epithelial cancers. *In vivo* study found an unexpected tumor-promoting role of NRF2 during early stage of skin tumorigenesis that was induced by virus [46]. NRF2 and its downstream genes were activated in the major precancerous human skin lesion [46]. Meanwhile, NRF2 overactivation is indicated to promote tumorigenesis in the liver of mice with loss of hepatic autophagy [47] or diethylnitrosamine administration [48]. Constitutive NRF2 activation promotes cancer initiation by conferring keratinocyte survival advantage (such as antiapoptosis) in adverse conditions [38, 49], as well as protumorigenic metabolic reprogramming toward anabolic glucose metabolism via the pentose phosphate pathway [46, 48]. The drug detoxifying function may sometimes mask the oncogenic role of NRF2 in cases of chemical carcinogenesis. The present study provides a direct proof that inhibiting Nrf2 expression during long-term exposure to a low-level arsenite prevents from malignant transformation. Thus, the role of NRF2 in homeostatic response and in the pathogenesis of cancer is more complex than expected from the basic concepts.

The oncogenic role of p62 has been reported in several cancers [12–14], including in the skin [15]. Silencing *p62* weakens malignant phenotypes of arsenite-transformed human lung bronchial epithelial BEAS-2B cells and human keratinocyte HaCaT cells, such as proliferation, migration, and clonogenicity [25, 43]. It is suggested that arsenic induces p62 accumulation due to disrupted autophagy. Once phosphorylated, p62 competes with NRF2 for binding to Keap1 [19], resulting in stabilization of NRF2 protein and subsequent activation of NRF2 downstream genes. Furthermore, p62 controls autophagy-induced degradation of Keap1 [50, 51] and is transcriptionally regulated by NRF2, thus forming a positive feedback loop with the NRF2 pathway. In the present study, we observed the augmented p62-NRF2 feedback loop in the arsenite-transformed keratinocyte model. The data suggest that protein degradation contributes to p62 protein accumulation in arsenite-exposed cells. This is consistent with the previous report that increased p62 mRNA level is insufficient for p62 protein aggravation, because the determinant factor of p62 protein level is autophagosomal-lysosomal proteolysis [52]. Upstream signaling of autophagy was also determined in this study. Activation of the mTORC1 pathway was observed in As-TM cells but not in acute arsenic-exposed cells (data not shown), which may be attributed to p62 accumulation [52, 53]. Data from acute exposure also suggest that arsenite inhibits autophagy efflux by interfering lysosome dysfunction, such as decreased expression of the key lysosome membrane proteins, LAMP1 and LAMP2 [54, 55]. The lysosome is a potential target organelle for arsenite toxicity and should be investigated in the future. Though alteration in response to acute arsenic exposure may not fully reflect mechanism underlying chronic exposure, determining the acute cellular response will greatly enhance our understanding of the early stages of arsenic carcinogenesis.

In summary, we have demonstrated that p62 accumulation resulting from inhibition of autophagic flux forms a positive feedback loop with the master regulator of antioxidative defense, NRF2, in response to the environmentally relevant dose of arsenite. Intervention of this loop may have significance in prophylactic strategy of arsenite-induced skin cancer and probably have implications in other arsenic-related cancers.

## Data Availability

The data used to support the findings of this study are included within the article.

## Supplementary Materials

Supplementary Materials Table S1: genes and primers for real-time RT-PCR.
