# Supplementary material for: Enhanced p62-NRF2 Feedback Loop due to Impaired Autophagic Flux Contributes to Arsenic-Induced Malignant Transformation of Human Keratinocytes
Source: Oxid Med Cell Longev. 2019 Oct 30;2019:1038932. doi: 10.1155/2019/1038932 (PMC6875345; doi:10.1155/2019/1038932)
Supplement: Supplementary Materials — Table S1: genes and primers for real-time RT-PCR. [file 1038932.f1.pdf]

Enhanced p62-NRF2 feedback loop due to impaired autophagic flux contributes to arsenic-induced malignant transformation of human keratinocytes

Xiafang Wu [#], Ru Sun [#], Huihui Wang, Bei Yang, Fang Wang, Hongtao Xu, Shimin Chen, Rui Zhao, Jingbo Pi, Yuanyuan Xu[*]

* Correspondence and request for materials should be addressed to Y.X. (email:

yyxu@cmu.edu.cn).

# The authors contribute equally to this work.

Table S1. Genes and primers for real time RT-PCR

Gene	GenBank Accession No.	Primers (5’→3’)
p62	NM_003900.5，NM_001142299.2，NM_001142298.2	Forward: GTGCACCCCAATGTGATCTG<br>Reverse: TGCACTTGTAGCGGGTTCCT
AKR1C1	NM_001353.6	Forward: TGGGAGGCCGTGGAGAA<br>Reverse: GGACACCCCGATGGACTTG
NQO1	NM_001025434.2,NM_001286137.2,NM_001025433.2, NM_000903.3	Forward: CAGCAGACGCCCCAATTC<br>Reverse: TGGTGTCTCATCCCAAATATTCTC
GCLC	NM_001498	Forward: GATGCTGTCTTGCAGGGAATG<br>Reverse: AGCGAGCTCCGTGCTGTT
GAPDH	NM_002046	Forward: CCTCCCGCTTCGCTCTCT<br>Reverse: CTGGCGACGCAAAAGAAGA
B-ACTIN	NM 001101.3	Forward: GAGCACAGAGCCTCGCCTTT<br>Reverse: TCATCATCCATGGTGAGCTGG
NRF2	NM_006164.4, NM_001145412.3, NM_001145413.3, NM_001313900.1, NM_001313901.1	Forward: AGCCCAGCACATCCAGTCA<br>Reverse: TGTGGGCAACCTGGGAGTAG

All primers are from Sigma-Aldrich.
